# Current perspectives on the mental health of UK military personnel and veterans

**DOI:** 10.1093/bmb/ldaf003

**Published:** 2025-05-16

**Authors:** Severija Juškaitė, Jennifer Stone, Neil Greenberg, Daniel Dyball, Nicola T Fear

**Affiliations:** King’s Centre for Military Health Research, King’s College London, Weston Education Centre, Cutcombe Road, London SE5 9RJ, UK; Academic Department of Military Mental Health, King’s College London, Weston Education Centre, Cutcombe Road, London SE5 9RJ, UK; Academic Department of Military Mental Health, King’s College London, Weston Education Centre, Cutcombe Road, London SE5 9RJ, UK; King’s Centre for Military Health Research, King’s College London, Weston Education Centre, Cutcombe Road, London SE5 9RJ, UK; King’s Centre for Military Health Research, King’s College London, Weston Education Centre, Cutcombe Road, London SE5 9RJ, UK; King’s Centre for Military Health Research, King’s College London, Weston Education Centre, Cutcombe Road, London SE5 9RJ, UK; Academic Department of Military Mental Health, King’s College London, Weston Education Centre, Cutcombe Road, London SE5 9RJ, UK

**Keywords:** United Kingdom, military personnel, mental health, veterans, positive psychology

## Abstract

**Introduction:**

This narrative review sets out to explore the current literature surrounding the mental health of serving personnel and veterans.

**Sources of data:**

UK literature published in peer-reviewed scientific journals and publicly available UK Ministry of Defence reports.

**Areas of agreement:**

Evidence suggests that common mental disorders are more prevalent in the UK serving and veteran personnel than in the general population. Risk factors include being female, adverse childhood events, combat exposure, and physical combat injury.

**Areas of controversy:**

Post-traumatic stress disorder rates are broadly similar between UK serving personnel and the general population, though certain groups are at increased risk, such as veterans who deployed to Iraq/Afghanistan in combat roles.

**Growing points:**

Understanding ways to maximize positive psychological outcomes, address poor sleep, and support those with combat injuries and chronic pain are key priorities for research/interventions. The prevalence of moral injury and military sexual trauma in the UK Armed Forces is currently unknown.

**Areas timely for developing research:**

A longitudinal study following recruits including those who are part of the Lesbian, Gay, Bisexual, Transgender, Queer/Questioning or other sexualities and gender identities (LGBTQ+) community, or who have a neurodivergent condition, from entry to post-service would enhance our understanding of how serving in the Armed Forces impacts the mental health and wellbeing.

## Introduction

The UK Armed Forces is a distinct occupational group undertaking a range of challenging roles that can be both gratifying and taxing, some of which may pose a risk to their mental health [1]. At the beginning of April 2024, the strength of the UK Armed Forces included 183 230 serving members, with 11 300 people joining the regular service in the past year and 15 730 personnel leaving service [2]. In the 2021 UK Census, 1 853 112 people in England and Wales indicated having previously served in the UK Armed Forces [3]. The Armed Forces covenant is a formal promise to support both serving personnel and veterans [4], which includes understanding, and caring for, their mental wellbeing.

Medical entry standards may disqualify individuals with certain mental health conditions, such as psychotic conditions including schizophrenia and delusional disorders, anxiety disorders including obsessive compulsive disorder, Post-Traumatic Stress Disorder (PTSD), dissociative disorders, somatoform disorders, eating disorders, and personality disorders, from joining the military [5]. Nevertheless, military personnel may possess pre-enlistment vulnerabilities that are associated with an increased risk of developing negative mental health outcomes. Examples include low educational attainment and adverse childhood experiences (e.g. experience of childhood abuse/neglect) [6]. In-service experiences may also increase the risk of negative mental health outcomes such as combat experiences including encountering small arms fire, exposure to improvised explosive devices [1], or experiencing a serious physical injury/illness on deployment [7], all of which can impact personnel’s emotional and physical health [8]. Military deployments can also incur disruptions to personnel’s sleep health [9], which is associated with both physical and mental health [10]. At the point an individual leaves service, the method by which they leave (e.g. unplanned or medically discharged) [11], the loss of military identity [12], leaving with a shorter total length of service [13], or being an early service leaver (i.e. leaving the Armed Forces with <4 years of service) [14] may be associated with negative mental health outcomes.

Focussing on pathology denies us the opportunity to understand the full breadth of human experience possible, such as factors associated with positive psychological functioning. One aspect is Post-Traumatic Growth (PTG), which can be defined as experiencing beneficial psychological changes following a traumatic event [15]. Moderate levels of PTG have been reported in military populations worldwide, with personnel from an ethnic minority background, with higher levels of spirituality, social support, and rumination, i.e. a re-examining of the beliefs/worldviews held relating to the traumatic event, more likely to endorse higher levels of PTG [16, 17].

### Aims

This narrative review will examine the rates of both negative mental health outcomes and PTG among UK Armed Forces serving personnel and veterans. This paper will also discuss pertinent risk factors, protective factors, and rates of help-seeking.

## Methods

To report on the mental health outcomes of the serving population, the review will use the 2023/24 Defence Statistics annual report on the rates and trends of UK Armed Forces mental health [18]. The report utilises data from individuals serving in the Armed Forces and is primarily gathered from the Ministry of Defence medical records. All regular military personnel have medical care provided or coordinated by the Defence Medical Services (DMS) [19]. After personnel leave the military, responsibility for healthcare reverts to the National Health Service (NHS). This includes some veteran-specific commissioned healthcare initiatives, such as Op Courage, which provides veterans with mental healthcare in England [20]. Serving personnel with mental health disorders are often initially seen by a military-employed General Practitioner, who can refer them to specialist mental health services, known as the Department of Community Mental Health (DCMH), if required [21]. DCMH staff are multidisciplinary including consultant psychiatrists, clinical psychologists, mental health nurses, occupational therapists, and mental health social workers. DCMHs provide all aspects of mental healthcare from triage, assessment, diagnosis, care coordination, and treatment. Inpatient mental healthcare for serving personnel is provided under contract by a consortium of eight NHS trusts to facilitate care close to an individual’s unit or home address [19].

To report on mental health outcomes among the serving and veteran population, the review will predominantly utilise data from two cohorts. The first cohort is the KCMHR military cohort study, which has been run by the King’s Centre for Military Health Research (KCMHR) since 2004 with the goal of assessing the health of military personnel who have deployed on operations in Iraq (Op TELIC) or Afghanistan (Op HERRICK) [22]. This cohort was conducted across multiple phases starting in 2004/6, phase 2 in 2007/9, phase 3 in 2014/16, and phase 4 in 2022/23. The second cohort is the ArmeD SerVices TrAuma and RehabilitatioN OutComE (ADVANCE) study, which investigates the long-term physical and psychosocial outcomes of UK military men who sustained serious physical combat injuries during deployment in Afghanistan [23]. The study compares an injured group with a frequency-matched, demographically similar group that sustained no such injuries. Additional independent studies from peer-reviewed journals are reported to supplement this data.

### Mental health

#### Common mental disorders

According to the 2023/24 Defence Statistics report, 0.7% of the UK serving population had been diagnosed with a mood disorder and 0.4% an anxiety or other neurotic disorder [18]; however, it is notable that these data refer only to personnel who have presented to the Defence Medical Services. Amongst those who sought help from a DCMH, depression was the most prevalent disorder (33.0%), followed by adjustment disorders (26.0%) and anxiety/other neurotic disorders (20.0%).

In the fourth phase of the KCMHR military cohort study, the rate of probable Common Mental Disorder (CMD) was 27.8% with the same rates between serving personnel and veterans; however, an overall increase in the rates of CMD over time was noted [24] with phase 3 showing the rate of CMD to be 21.9% [22].

In the ADVANCE Study, the rates of depression were 23.6% in the injured group (16.1% in the amputation related injury subgroup and 26.1% in the non-amputation related injury subgroup) and 16.8% in the comparison group [23]. The rates of anxiety were 20.8% in the injured group (14.1% in the amputation related injury subgroup and 23.1% in the non-amputation related injury subgroup) and 13.5% in the comparison group [23].

Data from a primary healthcare cohort study, which reports on data from medical records in a sample of 2449 veterans living in the Northwest of England, found that 38.0% of veterans in Northwest England had a medical diagnosis indicating a mental health disorder [25]. Depression was the most prevalent disorder at 17.8%, followed by alcohol misuse (17.3%) and anxiety (15.0%) [25].

An investigation into the KCMHR military cohort study found that the odds of probable CMD were over double in UK Armed Forces serving personnel when compared to employed civilians [adjusted Odds Ratio (OR) = 2.3, 95% Confidence Interval (CI) = 2.0–2.6] [26].

#### Post-traumatic stress disorder

According to the 2023/24 Defence Statistics report, 0.2% of the UK serving population had been diagnosed with Post-Traumatic Stress Disorder (PTSD); however, it is notable that these data refer only to personnel who have presented to the Defence Medical Services [18]. Amongst those who sought help from a DCMH, the prevalence of PTSD was 11.0%.

In the fourth phase of the KCMHR military cohort study, the overall rate of probable PTSD was 9.4%, with significantly higher rates among veterans (10.5%) compared to serving personnel (7.4%) [24]. The prevalence of probable PTSD has increased over time, with 4.0% reported in Phases 1 and 2 and 6.2% in Phase 3 [22]. Deploying to Iraq/Afghanistan was not associated with probable PTSD for regular personnel who were still serving in the UK Armed Forces (7.5%) compared to serving personnel who did not deploy there (7.0%) [24]. However, veterans who, when in service, had deployed to Iraq/Afghanistan had higher rates of probable PTSD (12.6%) compared to veterans who had not deployed there (7.6%) [24]. Veterans who deployed to Iraq/Afghanistan in a combat role had the highest rates of PTSD (18.4%) [24].

In the ADVANCE Study, the rates of probable PTSD were 16.9% in the injured group (9.9% in the amputation related injury subgroup and 19.3% in the non-amputation related injury subgroup) and 10.5% in the comparison group [23].

A primary healthcare cohort study found that 3.4% of veterans living in Northwest England had been diagnosed with PTSD [25].

The trajectory of PTSD symptoms over a 12-year period in UK military personnel was investigated in the KCMHR military cohort study [27]. Overall, the majority of the UK Armed Forces did not experience symptoms indicative of probable PTSD (71.3% experienced no/low symptoms, and 17.3% experienced slightly elevated symptomology). 4.6% of personnel experienced ‘improving’ symptoms, i.e. they experienced probable PTSD symptomology at one time point, which reduced to a subclinical threshold over the study period. 4.9% of personnel experienced ‘worsening’ symptoms, i.e. they experienced subclinical PTSD symptomology at one point, which increased over time to meet probable PTSD caseness. Finally, 1.8% of personnel experienced ‘chronic’ symptoms, i.e. their PTSD symptomology was consistently above threshold for probable PTSD caseness across the study period. Veterans represented a greater percentage of personnel in the chronic (2.3% vs. 1.2%) and worsening (5.6% vs. 4.2%) classes compared to still-serving personnel.

#### Post-traumatic growth

An analysis of the KCMHR military cohort Phase 3 data showed that 34.8% of women and 30.8% of men reported moderate to high levels of PTG following an operational deployment to Iraq/Afghanistan [28]. The study found that probable CMD was associated with no/very low levels of PTG. There was a curvilinear relationship between probable PTSD and PTG: levels of PTG increased alongside severity of PTSD symptoms up to a certain point, after which, as PTSD symptoms became more severe, levels of PTG decreased. Further analysis indicated that military personnel who reported experiencing a greater number of combat experiences, a greater belief of being in danger during deployment, better general health, lower alcohol use, and reservists (compared to regular personnel) were more likely to report moderate to high levels of PTG. There were notable gender differences: leaving service and being single were only associated with PTG among female personnel, whereas overall perceived health was only associated with PTG among male personnel [28].

In the ADVANCE study, 45.4% of UK Armed Forces personnel who sustained amputation injuries in Afghanistan, 34.1% of those who sustained non-amputation related injuries and 28.0% of demographically similar personnel who sustained no such injuries reported a large degree of PTG [29]. Further, pain, depression, and PTSD mediated the association between sustaining a serious physical combat injury and experiencing a large degree of PTG.

### Risk and protective factors of negative mental health outcomes in UK serving personnel and veterans

#### Sociodemographic

As of April 2024, women made up 11.7% of the UK regular Armed Forces [30]. Women are more commonly diagnosed with CMD than men [31] in both civilian and military groups [26]. Within the serving population, a greater percentage of women attended DMS for mental health–related reasons compared to men [18]. In a primary healthcare cohort study, women veterans living in Northwest England had higher rates of mental health disorder diagnoses than men (44.0% vs. 37.5%) [25]. It is unclear if this difference is due to women being more likely to experience mental health disorders or due to women being more likely to seek help and get a diagnosis [32].

In the PTSD symptom trajectory analysis of the KCMHR military cohort study, those who held a lower rank, had served in the Army, reported probable alcohol misuse, adverse childhood experiences, and inconsistent post-deployment social or military support were more likely to be represented in the worsening or chronic PTSD symptomology classes [27].

A retrospective cohort study of over 78 000 veterans living in Scotland explored the mental health outcomes of those who joined service younger than 17.5 years old (termed junior entrants) compared to those who were between 20 and 25 years of age at the time of entry [33]. The study found no evidence that junior entrants had any difference in likelihood of having any mental health diagnosis or a specific PTSD diagnosis compared to those who entered service at 20–25 years old or civilians with no record of military service. The KCMHR military cohort study investigated junior entrants compared to older entrants (e.g. older than 17.5 years old) [34]. It was found that junior entrants were no more likely to deploy to Iraq/Afghanistan, but, if they did deploy, they were more likely to do so in a combat role. No differences in the odds of reporting probable CMD, PTSD, self-harm, or alcohol misuse were observed in junior entrants compared to older entrants. Junior entrants were also no different from older entrants in their odds of experiencing trouble with the law, unemployment, or financial difficulties after leaving service. However, in a subsample of those who joined the Armed Forces after April 2003 (i.e. near the start of the Iraq War), junior entrants were more likely to endorse multiple somatic symptoms (adjusted OR = 1.5, 95% CI 1.0–2.2), lifetime self-harm (adjusted OR = 2.1, 95% CI 1.2–4.0), and alcohol misuse (adjusted OR = 1.8, 95% CI 1.2–2.9) compared to older entrants.

#### Combat experiences and role

In Phase 3 of the KCMHR military cohort study, military personnel who reported high levels of combat experiences had nearly three times the odds of reporting probable PTSD (OR = 2.7, 95% CI = 1.6–4.6) and were more likely to report probable CMD (OR = 1.5, 95% CI = 1.2–1.8) compared to those who reported low levels of combat experiences [1]. Personnel with high levels of combat experience also had three times the risk of reporting comorbid mental disorders [multinominal relative risk = 3.2, 95% CI = 2.2–4.7] compared to those with low levels of combat experiences, relative to the risk of reporting no disorders [1]. For veterans, holding a combat role whilst in service was significantly associated with probable PTSD and CMD compared to those who had served in a combat service-support role [22]. In the PTSD symptom trajectory analysis of the KCMHR military cohort study, personnel with combat experiences such as ‘being in proximity of wounding/death’ and ‘experience of violent combat’ were more likely to report chronic levels of PTSD symptomology [27].

#### Sleep

Sleep dysfunction is associated with higher rates of physical disease, mental illness, and general mortality [10]. Sleep also has a bidirectional relationship with mental illness, whereby sleep dysfunction increases the risk of mental illness and mental illness increases the risk for sleep dysfunction [10]. In a study of UK Armed Forces personnel who were potentially exposed to traumatic combat events while deployed to Afghanistan, it was found that greater time spent deployed was associated with multiple dimensions of poor sleep health post deployment, such as sleep interfering with daily functioning, losing sleep over worry, sleep dissatisfaction, and having trouble falling or staying asleep [9]. Poor sleep health was associated with mental health problems, such as probable CMD and PTSD, and poor sleep health post-deployment was predictive of reporting mental health problems at follow-up but not of alcohol misuse [9].

#### Social support

Social support is a protective factor against mental ill health, and, in the military context, both PTG and unit support were found to be associated with social support [35]. Following deployment to Iraq/Afghanistan, perceived social support was associated with better mental health outcomes and psychological functioning in UK Armed Forces personnel [35]. Further research on serving personnel and veterans explored the relationship between CMD, social participation, and social networks and found that compared to serving personnel, veterans were more likely to report lower levels of social participation [36]. Increased risk of experiencing CMD symptoms in veterans may be attributed to low levels of social integration; however, this did not account for the PTSD symptoms. For veterans, maintaining social contacts with actively serving personnel was associated with greater odds of reporting both probable CMD and PTSD compared to personnel still in service [36].

The association between marital status and mental health was investigated in Phase 4 of the KCMHR military cohort study [24]. Those not in a relationship (single/ex-relationship) had rates of 17.1% and 36.5% for PTSD and CMD, respectively [24]. Those in a relationship had rates of 8.0% and 26.3% for PTSD and CMD, respectively [24].

#### Help-seeking

Data from the annual Defence Statistics report indicates that 9.8% of UK Armed Forces personnel were seen by DMS for a mental health-related reason in 2013/14, which increased to 13.0% in 2023/24 [18]. Approximately 2.0% of serving personnel received specialist mental health treatment from a DCMH in 2023/24, which is reported to be lower than the rates in the general population who accessed secondary mental health services in 2022/23 (6.0%) [18], although it is notable that many people with a significant history of poor mental health would not have been able to join the military.

In an extension of the KCMHR military cohort study investigating help-seeking in UK military serving personnel/veterans who reported a stress/emotional problem in the past 3 years, only 7.0% of participants did not seek any form of help [32]. Eighty-six percent had used informal sources of support, 55.0% received help from formal medical services and 46.0% from formal non-medical sources. UK service personnel and veterans were found to have good awareness of and willingness to access formal medical sources of support. However, veterans had a lower awareness of services set up to specifically help veterans compared to other sources of support (formal medical or informal) and had a lower willingness to access those services [37]. It has been suggested that veterans might be uncomfortable using civilian services as they may feel like civilian healthcare providers might not understand their military experiences [38]. Women veterans also reported that the lack of recognition of their veteran status and perceived negative gender stereotyping by civilian healthcare professionals had discouraged them from seeking help post-service [38]. This is further supported by a qualitative study, which found that female veterans might experience unique barriers to mental health care in addition to the barriers experienced by veterans in general, such as ‘access barriers, lack of understanding from professionals, gender-related discrimination, mental health stigma, and sexual orientation-related discrimination’ (p. 151) [39].

## Discussion

This review discusses the negative mental health outcomes and positive psychological functioning of UK Armed Forces personnel (serving and veterans) as well as the interplay of risk and protective factors influencing these outcomes. Current data indicate that the rates of CMD are higher in military than general populations [26], while the rates of PTSD are broadly similar [18, 25]. However, specific groups, such as veterans with high levels of combat exposure [22], or pre-enlistment vulnerabilities, such as adverse childhood experiences [6], report greater rates of PTSD compared to the general population.

### Areas of agreement

CMDs are more prevalent in military personnel (27.8%) [24] compared to the general population (17.0%) [40] and compared to employed civilians [26]. No significant differences exist in rates of CMD between veterans and serving personnel [24]. It has been suggested that the differences observed between military and other civilian occupations could be due to reasons such as time spent away from family and home [31], moving every 2–3 years, uncertainty around being deployed [26], or due to the high prevalence of adverse childhood experiences in the military population [6]. Rates of CMD in the general population are usually based on household studies, while military rates are based on occupational studies. This is important to note, as occupational studies have been found to be susceptible to response bias due to a framing effect, whereby participants might be more likely to take part in a study or report greater severity of symptoms if they have a perceived grievance with the occupation [26]. Indeed, when rates of CMD in the military are compared to rates of CMD in other professions such as teachers, social workers, and academics, rates of CMD tend to be lower in the military [41].

CMD is more prevalent in women compared to men in both civilian and military populations [26, 31]. Although women who served in the Armed Forces are more likely to access formal mental health support, they are less likely to access informal sources of support compared to men [42]. Increased risk of specific military experiences, such as Military Sexual Trauma (MST), might also be partly responsible for differences in rates of CMD or greater demands and stressors at home [31].

The Defence Statistics annual review draws its data from the medical records of serving personnel; thus, overall rates of help-seeking could be underrepresented, as some serving personnel might seek help outside the military and indeed some individuals might not seek help at all due to not recognizing the signs of mental illness or due to reluctance to seek formal support due to mental health stigma or fear of negative occupational outcomes [32]. Most serving and veteran personnel seek help when facing emotional or stress-related problems (93.0%) [32]. However, veterans appear to have limited awareness of and willingness to access professional/clinical services specifically designed for them [37]. The authors discuss whether this could be due to veterans feeling confused about the various services that are available to them once they transition into civilian life. It has also been suggested that veterans might not want to seek help due to internalized, public, or anticipated stigma as well as concerns about sharing confidential information, distrusting the healthcare system, and the belief that the problem must be ‘severe’ enough to seek treatment [43]. Additionally, it has been suggested that veterans might be uncomfortable using civilian services as they feel like civilian healthcare providers might not understand their military experiences [38]. Future research could investigate the reasons behind the reluctance of veterans to use veteran-specific services and explore ways to increase awareness and uptake.

### Areas of controversy

The media often portrays PTSD as prevalent in the military [44]; however, amongst serving personnel, rates of PTSD are broadly similar to those observed in the general population (4.4%) [40], ranging from 0.2% [18] to 7.4% [24]. Rates in veterans range from 3.4% [25] to 10.5% [24], with veterans who deployed when in service to Iraq or Afghanistan in a combat role (18.4%) [24] or experienced a combat injury (16.9%) [23] having elevated rates of PTSD. The disparity between PTSD rates in serving personnel and veterans could be due to those who experience PTSD leaving service as a result of their symptoms, either voluntarily or involuntarily (e.g. medical discharge) or due to delayed onset of PTSD after discharge [45].

### Growing points

Positive psychology research, particularly in areas like PTG, is becoming more common in the military context, and the protective role that positive psychological functioning [28, 29] might provide against mental illness requires further investigation.

Moral injury is another growing area of military research. Moral injury is defined as ‘enduring psychosocial, spiritual or ethical harms that can result from exposure to high-stakes events that strongly clash with one’s moral beliefs’ [46, 47], Exposure to potentially morally injurious experiences has been linked to subsequent adverse mental health outcomes, such as suicidal ideation and anxiety disorders [47]. Furthermore, a pilot study highlighted the need for a validated treatment approach as well as greater awareness of moral injury in veterans’ clinical teams and highlighted the challenges clinicians face when treating veterans with moral injury [48]. The prevalence of moral injury in the UK Armed Forces remains unknown, and future research should address this in both serving and veteran populations.

Sleep disorders in military personnel are a growing health concern [49]. Deploying for longer periods of time has been linked to disordered sleep [9]. Given the evidence linking sleep health to mental and physical health [10], interventions that address sleep problems in serving and veteran populations have the potential to positively affect both physical and mental wellbeing. One suggested strategy involves leaders promoting healthy sleep practices by educating their units about sleep hygiene and endorsing strategies like sleep banking or tactical napping [49].

Measuring chronic pain is challenging due to the difficulty in quantifying and averaging pain and heterogeneity in definitions of pain [50]. There is a strong correlation between PTSD and chronic pain in veteran populations; however, research on the prevalence of PTSD and chronic pain comorbidity in UK veterans is currently limited [51]. The ADVANCE study found an association between negative mental health outcomes, such as higher rates of depression, anxiety and PTSD, and pain [52]. In fact, rates of PTSD were four times greater in the injured personnel who reported moderate/severe pain compared to injured personnel who reported no/mild pain [52]. Thus, research on chronic pain, both on its own and comorbid with adverse mental health, is an area of growing interest in military research.

Another emerging area of research surrounds MST. Currently, the rates of MST are unknown in the UK serving and veteran populations. Most research on MST is conducted on women; however, although women are more likely to experience MST [53], it is not a women-only issue and experiences of people across the gender spectrum should be studied.

Emerging evidence on combat injuries highlights the relationship between physical and mental health, raising new research questions. Notably, UK Armed Forces personnel who deployed to Afghanistan and sustained non-amputation injuries have been found to experience greater rates of negative mental health outcomes than those who sustained amputation injuries or demographically similar personnel with no serious physical combat injuries [23]. Longitudinal research is needed to understand whether rates of probable mental health disorders change as the population ages and age-related physical conditions become more prevalent [54]. Clinicians who work with serving or veteran personnel who deployed on operations should enquire about mental health and consider the impact of less visible injuries.

### Areas of timely developing research

In a 2023 review on UK Armed Forces incentivization, it was stated that the Armed Forces might need to amend their medical entry standards in light of the changing UK population, namely, in regard to neurodiversity, fitness standards, residency, and nationality [55]. There are currently no known cohorts set up to assess the mental health of serving personnel from these demographics as they enter and transition through military service. There is also a need for research into other currently underrepresented groups such as those who are part of the Lesbian, Gay, Bisexual, Transgender (LGBT) community [56, 57]. A longitudinal study following a representative sample of recruits including those who are part of the LGBTQ+ community, or who have a neurodivergent condition, from entry to post-service would enhance our understanding of how serving in the Armed Forces impacts the mental health and wellbeing and whether military specific or post-service experiences carry reduced/enhanced risk for people from these demographics.

### Strengths and limitations

A key strength of this review is its narrative approach, which allows for an in-depth exploration of mental health issues in the UK serving and veteran population through a collection of key studies and reports. The studies reviewed utilise various data collection methods—medical records in the annual Defence Statistics report [18] and self-report data in the KCMHR military cohort [22, 24] and the ADVANCE cohort study [23]. The range of studies provide valuable clinical and research perspectives on the mental health of the UK Armed Forces. However, it is important to note that due to the differences in collection methods (e.g. screening questionnaires and medical records), it is difficult to make direct comparisons between the studies.

This review focuses on select areas of mental health rather than covering all possible outcomes; thus, areas such as serious mental illness (e.g. schizophrenia), suicide, self-harm, gambling, and alcohol misuse have not been addressed in the present paper. These outcomes were outside of the scope of the current review, and recent reviews available elsewhere address suicide and self-harm within the UK Armed Forces [13, 58]. The current review is also based exclusively on UK Armed Forces data and so lacks any direct comparisons with data from other countries.

## Conclusions

While some studies suggest the rates of CMD tend to be greater in the military compared to civilian populations, this may not be the case if military personnel are compared with other civilian occupational groups. Although the UK media presents PTSD as a particular problem for the Armed Forces, the available evidence indicates that rates of PTSD in serving personnel are comparable to the general population. Elevated rates of PTSD have, however, been observed in specific groups of veterans, such as those who, when in service, have deployed to Iraq or Afghanistan in a combat role or sustained combat injuries. Clinicians should remain aware of pre-enlistment vulnerabilities as well as military experiences that may be associated with increased risk of poor mental health outcomes when treating serving personnel and veterans.

## Data Availability

No new data were generated or analysed in support of this review.

## Notes

Conflict of interest: D. Dyball and N. Greenberg declare that they have no conflicts of interest. N. T. Fear and S. Juškaitė are part funded by a grant from the UK Ministry of Defence. N. T. Fear is also a trustee (nonpaid) of a charity supporting the wellbeing of service personnel, veterans, and their families. J. Stone is a serving member of the British Army. The views expressed are those of the authors and not necessarily those of the UK Ministry of Defence.
